# Insertion/deletion hotspots in the Nsp2, Nsp3, S1, and ORF8 genes of SARS-related coronaviruses

**DOI:** 10.1186/s12862-022-02078-7

**Published:** 2022-10-28

**Authors:** Tetsuya Akaishi, Kei Fujiwara, Tadashi Ishii

**Affiliations:** 1 Department of Education and Support for Regional Medicine, Tohoku University, Seiryo-machi 1-1, Aoba-ku, 980-8574 Sendai, Miyagi Japan; 2 COVID-19 Testing Center, Tohoku University, Sendai, Japan; 3 Department of Gastroenterology and Metabolism, Nagoya City University, Nagoya, Japan

**Keywords:** Bat coronavirus, insertion/deletion (indel), Indel hotspot, Pangolin coronavirus (PCoV), Severe acute respiratory syndrome coronavirus 2 (SARS-CoV-2)

## Abstract

The genome of severe acute respiratory syndrome coronavirus 2 (SARS-CoV-2) contains many insertions/deletions (indels) from the genomes of other SARS-related coronaviruses. Some of the identified indels have recently reported to involve relatively long segments of 10–300 consecutive bases and with diverse RNA sequences around gaps between virus species, both of which are different characteristics from the classical shorter in-frame indels. These non-classical complex indels have been identified in non-structural protein 3 (Nsp3), the S1 domain of the spike (S), and open reading frame 8 (ORF8). To determine whether the occurrence of these non-classical indels in specific genomic regions is ubiquitous among broad species of SARS-related coronaviruses in different animal hosts, the present study compared SARS-related coronaviruses from humans (SARS-CoV and SARS-CoV-2), bats (RaTG13 and Rc-o319), and pangolins (GX-P4L), by performing multiple sequence alignment. As a result, indel hotspots with diverse RNA sequences of different lengths between the viruses were confirmed in the Nsp2 gene (approximately 2500–2600 base positions in the overall 29,900 bases), Nsp3 gene (approximately 3000–3300 and 3800–3900 base positions), N-terminal domain of the spike protein (21,500–22,500 base positions), and ORF8 gene (27,800–28,200 base positions). Abnormally high rate of point mutations and complex indels in these regions suggest that the occurrence of mutations in these hotspots may be selectively neutral or even benefit the survival of the viruses. The presence of such indel hotspots has not been reported in different human SARS-CoV-2 strains in the last 2 years, suggesting a lower rate of indels in human SARS-CoV-2. Future studies to elucidate the mechanisms enabling the frequent development of long and complex indels in specific genomic regions of SARS-related coronaviruses would offer deeper insights into the process of viral evolution.

## Background

The genome of severe acute respiratory syndrome coronavirus 2 (SARS-CoV-2) has been reported to incorporate multiple relatively long and complex insertion/deletion (indel) mutations from the SARS-CoV genome [1, 2], and some of the indels are believed to have played critical roles in the development of SARS-CoV-2 ^3^. These non-classical complex indels that cannot be simply explained by the conventional sole insertions or deletions are concentrated in non-structural protein 3 (Nsp3), the S1 domain of the spike (S), and open reading frame 8 (ORF8). Some of these relatively long and complex indels have been suggested to be developed through a novel type of mutation, realizing a thorough replacement of relatively long RNA sequences of the involved regions [4]. A previous study has demonstrated that the mutation profiles of SARS-related coronaviruses are largely different from those of other RNA and DNA viruses [5], and such non-classical indels are not seen in all types of virus other than coronaviruses. Currently, the frequency and distribution of such relatively long and complex indels in SARS-related coronaviruses other than SARS-CoV and SARS-CoV-2 remain unclear. To clarify the distribution of indel hotspots and the presence of such non-classical complex indels in a broad spectrum of SARS-related coronaviruses in the natural environment, the present study compared the whole genome sequences of viruses from humans (SARS-CoV and SARS-CoV-2), bats (RaTG13 from China and Rc-o319 from Japan), and pangolins (GX-P4L).

## Results

### Mutations in SARS-CoV and PCoV_GX-P4L

**Fig. 1:**
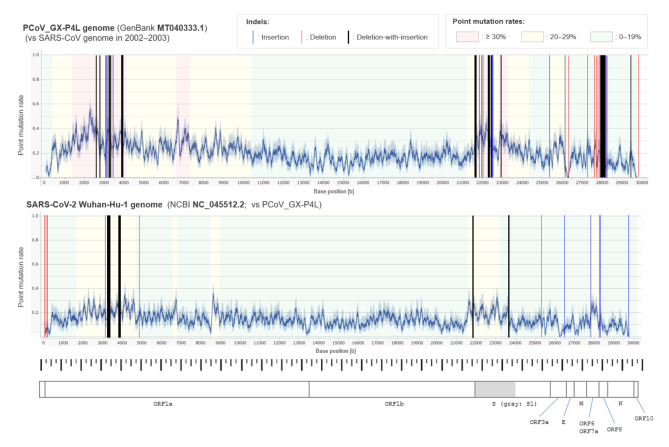
**Distribution of point mutation rate and indels in the genomes of PCoV_GX-P4L and SARS-CoV-2** The base position-oriented point mutation rate, together with the locations of indels (solid bars), across the genomes of PCoV_GX-P4L and SARS-CoV-2 are shown, compared with the reference genome of SARS-CoV from 2002–2003. The point mutation rate was calculated as the rolling average of the point mutation rate in the nearest ± 50 bases at each base position after excluding the base positions with indels. The blue areas around the line graphs represent 95% confidence intervals for the point mutation rates. Indels were categorized into the following three general types: insertions (blue bar), deletions (red bar), and deletions-with-insertions (black bar). The width of each bar is proportional to the length of the consecutive bases involved in each indel site E, envelope; M, membrane; N, nucleocapsid; ORF, open reading frame; PCoV_GX-P4L, pangolin coronavirus GX-P4L; S, spike; SARS-CoV, severe acute respiratory syndrome coronavirus

The pangolin coronavirus GX-P4L genome was 77.1% identical to the SARS-CoV genome obtained in 2003. The mutations comprised 5751 bases (84.3%) point mutations and 1075 bases (15.7%) with indels. There were 34 indel sites, and the sizes of the indels ranged from 1 to 327 consecutive bases. In the 34 indel sites, 17 were based on relatively long and complex indels that cannot be simply explained by sole insertions or deletions (e.g., with replaced sequences adjacent to gaps). These non-classical complex indels that replace a certain length of sequences, often with changed base lengths, are called deletion-with-insertion in the present study. These non-classical complex indels were concentrated in Nsp3 of the open reading frame 1a (ORF1a) gene and the S1 domain of the S gene. The distribution of the point mutation rate and indels across the PCoV_GX-P4L genome is shown in the upper panel of Fig. 1. For reference, the distributions of the point mutation rate and indels in the genomes of SARS-CoV and SARS-CoV-2 have been previously reported [4].

### Mutations in SARS-CoV-2 and PCoV_GX-P4L

To confirm the presence of non-classical complex indels between a pair of more recently sequenced virus lineages with lower point mutation rates, the genome sequences of PCoV_GX-P4L and SARS-CoV-2 obtained in December 2019 were further compared. The genome of PCoV_GX-P4L was 84.9% identical to that of SARS-CoV-2. The mutations comprised 4221 bases (93.7%) with point mutations and 282 (6.3%) with indels. There were 15 indel sites, and four of them were based on complex deletions-with-insertions. Two of the four deletions-with-insertions were located in Nsp3, and the other two in the S1 gene. The sizes of the bases involved in the four deletion-with-insertion sites ranged from 20 to 135 consecutive bases. The distribution of the point mutation rate and indels across the SARS-CoV-2 genome is shown in the lower panel of Fig. 1.

### Indel hotspots based on multiple sequence alignment

There were 5–10 gene sites with different types of indels between the genomes of SARS-CoV, SARS-CoV-2, bat Rc-o319, and pangolin GX-P4L, which are considered indel hotspots. These hotspots were located in the Nsp2 gene (approximately 2500–2600 base positions in the overall 29,900 bases), Nsp3 gene (3000–3300 and 3800–3900 base positions), N-terminal domain of the spike protein (21,500–22,500 base positions), and ORF8 gene (27,800–28,200 base positions). The indel hotspot in Nsp2 corresponded to amino acid positions 564–597 of the overall 638 amino acid residues in Nsp2. The indel hotspots in Nsp3 corresponded to amino acid positions 93–193 and 359–393 of the overall 1945 amino acid residues in Nsp3.

**Fig. 2:**
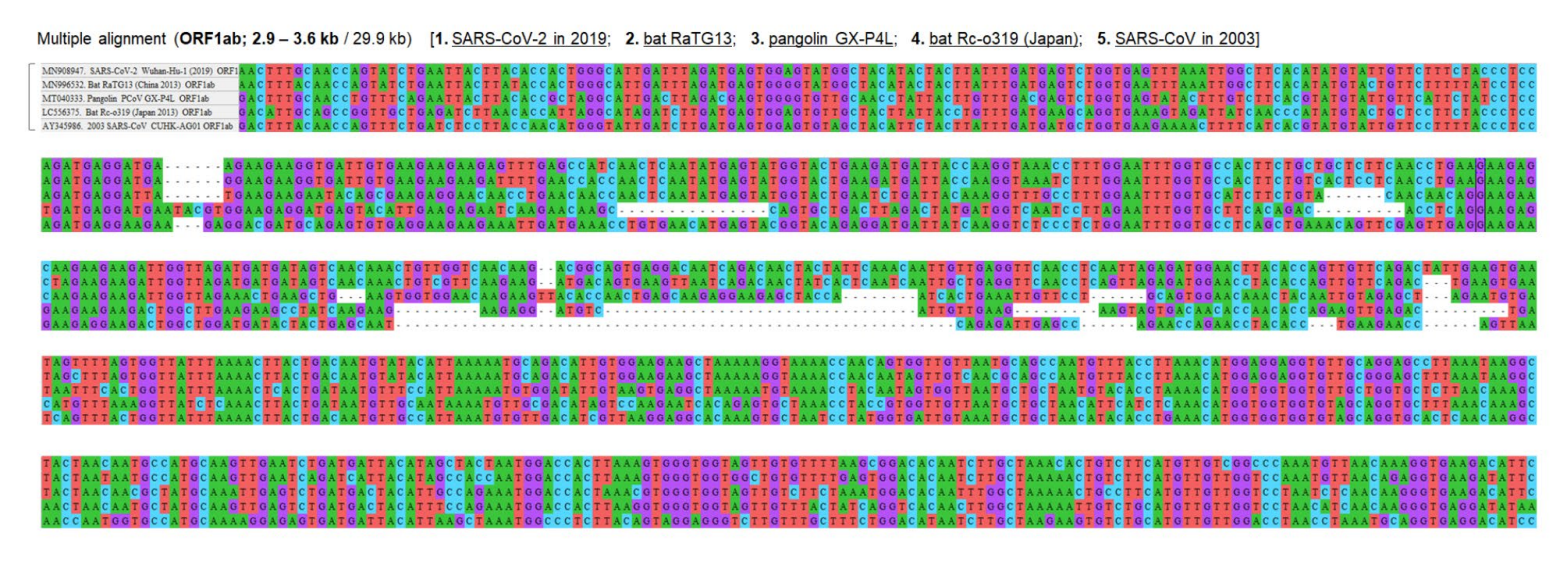
**Multiple sequence alignment in the Nsp3 of OFR1ab gene** Different patterns of indels were observed in this indel hotspot located in the Nsp3 gene of the ORF1ab gene (approximately 3.0–3.3 kb of the complete 29.9 kb sequence). The observed patterns of indels suggest that different types of indels frequently occur and sometimes overlap at the same base position in the indel hotspot, creating diverse patterns of indels between virus species. In addition to the shown indel hotspot, there are two other indel hotspots in the ORF1ab gene (approximately at 2.5–2.6 kb and 3.0–3.3 kb of the complete 29.9 kb sequence) Nsp3, non-structural protein 3; ORF1ab, open reading frame 1ab; SARS-CoV, severe acute respiratory syndrome coronavirus

**Fig. 3:**
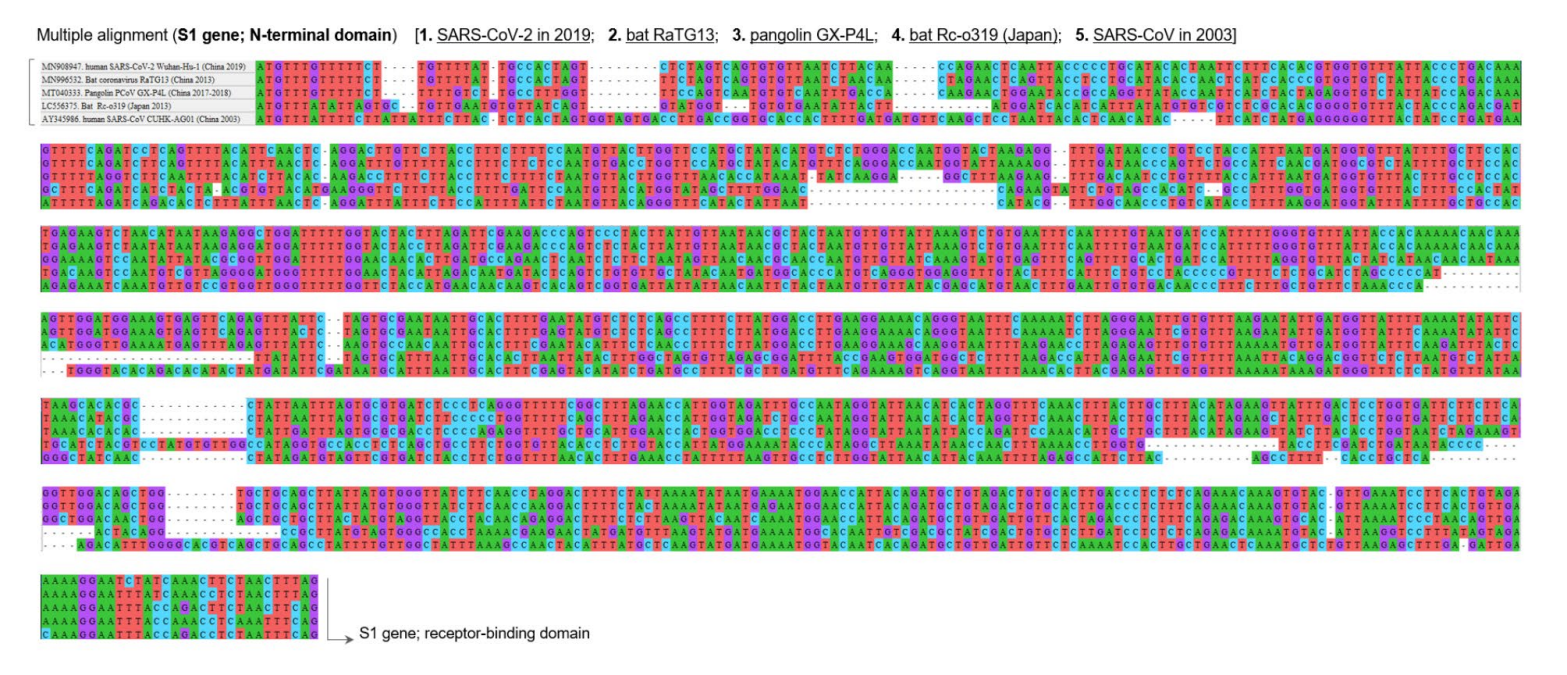
**Multiple sequence alignment in the N-terminal domain of S1 gene** Distribution of indels in the N-terminal domain of the S1 gene in the five SARS-related coronaviruses is shown. Based on the frequency and length of the indels, this domain is regarded as another site of indel hotspots. The frequency of indels in the N-terminal domain is significantly higher than that in the receptor-binding domain S, spike; SARS-CoV, severe acute respiratory syndrome coronavirus

**Fig. 4:**
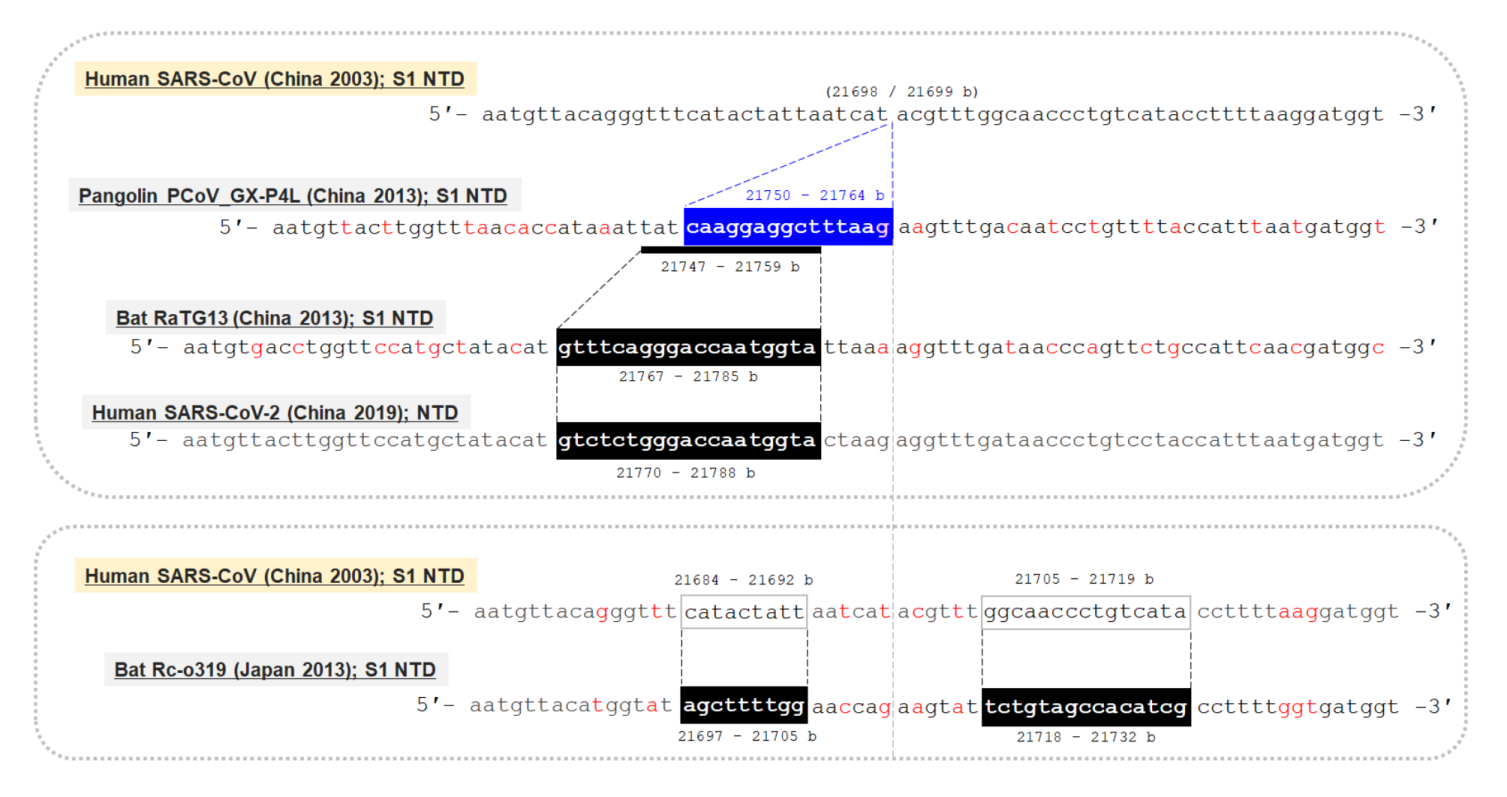
**An actual hotspot for indels in the N-terminal domain of the S gene** The actual hotspot site for indels in the N-terminal domain of the S gene is shown, which corresponds to the middle of the second row in Fig. 3. All genomes of human SARS-CoV-2, bat Rc-o319, and pangolin GX-P4L incorporated different types of indels than that of the genome of SARS-CoV in 2003. These findings indicate that this genome site is a hotspot for indels. The indels in the genome of bat Rc-o319 are considered to have developed based on a novel mutation type, deletions-with-insertions b, base; PCoV_GX-P4L, pangolin coronavirus GX_P4L; S, spike gene; SARS-CoV-2, severe acute respiratory syndrome coronavirus 2

**Fig. 5:**
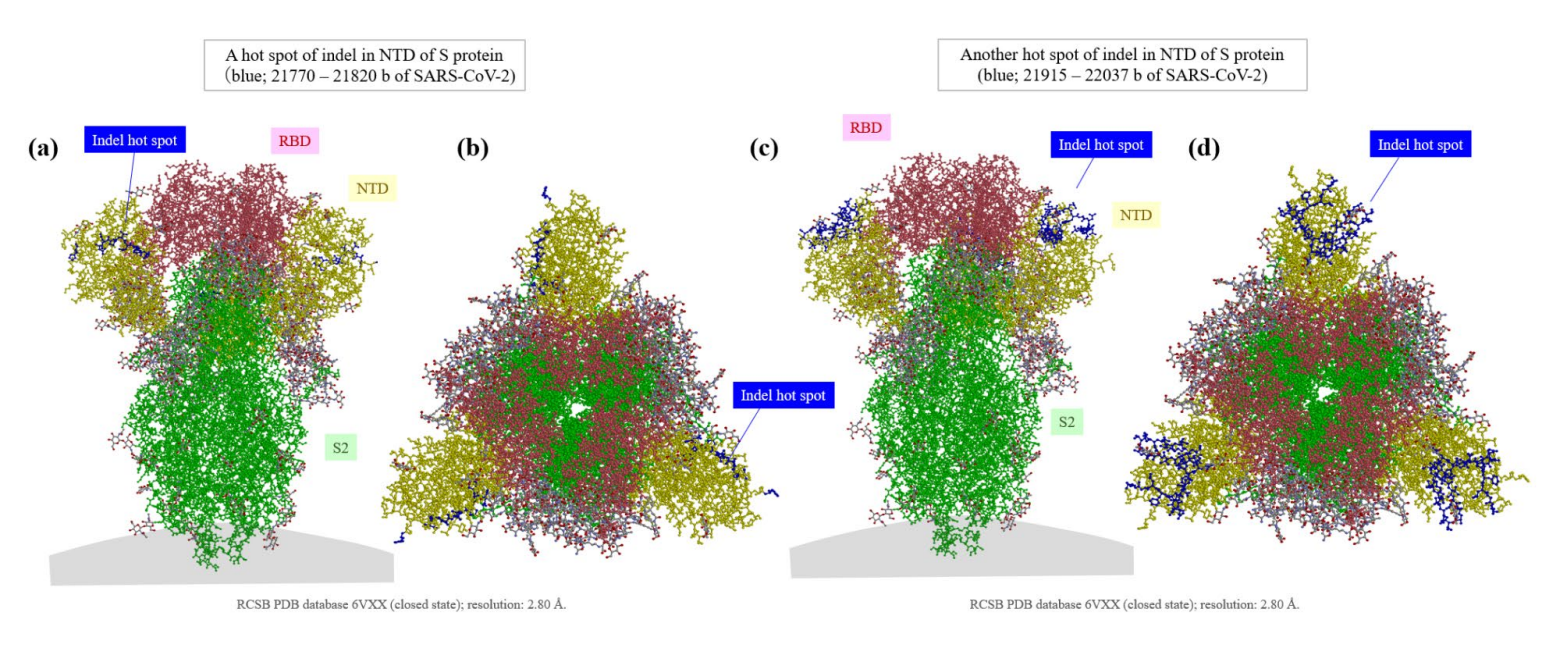
**Two hotspots for indels on the molecular structure of the SARS-CoV-2 S protein** Two hotspots for indels in the N-terminal domain of the S protein are displayed on the 3D molecular structure of the S protein of SARS-CoV-2. The first hotspot is located at base numbers 21,770–21,820 (a: axial view, b: upper coronal view) and the second hotspot is located at base numbers 21,915–22,037 (c: axial view, d: upper coronal view). Both hotspots were located on the surface of the N-terminal domain of the S protein NTD, N-terminal domain; RBD, receptor-binding domain; S, spike; SARS-CoV-2, severe acute respiratory syndrome coronavirus 2

**Fig. 6:**
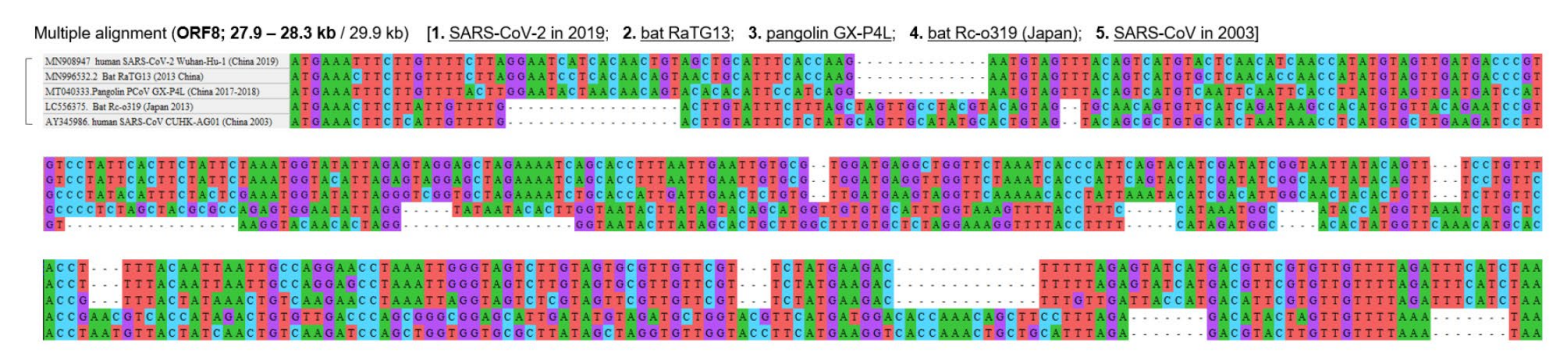
**Multiple sequence alignment in the ORF8 gene** Distribution of indels in the ORF8 gene in the five SARS-related coronaviruses is shown. Multiple in-frame and frameshift mutations are frequently observed across the ORF8 gene ORF, open reading frame; SARS-CoV, severe acute respiratory syndrome coronavirus

The result of multiple alignment at one of the indel hotspots in the Nsp3 gene is shown in Fig. 2, and that in the N-terminal domain of the S gene is shown in Fig. 3. Although the indel patterns were similar between the Wuhan-Hu-1 and RaTG13 genomes, the other pairs of SARS-related coronaviruses showed different indel patterns, all with different lengths and positions. A close look at the sequences in one actual site of such an indel hotspot in the N-terminal domain of the S gene is shown in Fig. 4. Collectively, these findings show that the pangolin GX-P4L, bat RaTG13, SARS-CoV-2, bat Rc-o319, and SARS-CoV genomes incorporate different types of indels (classical sole insertions, sole deletions, or non-classical complex indels) with different lengths in this genome area. Including this hotspot, two hotspots for indels in the N-terminal domain of the S protein are shown in the three-dimensional (3D) structure of the S protein in Fig. 5. Both hotspots are located on the surface of the N-terminal domain. The result of multiple alignment in the ORF8 gene is shown in Fig. 6.

### Phylogenetic trees in ORF1ab and S genes

**Fig. 7:**
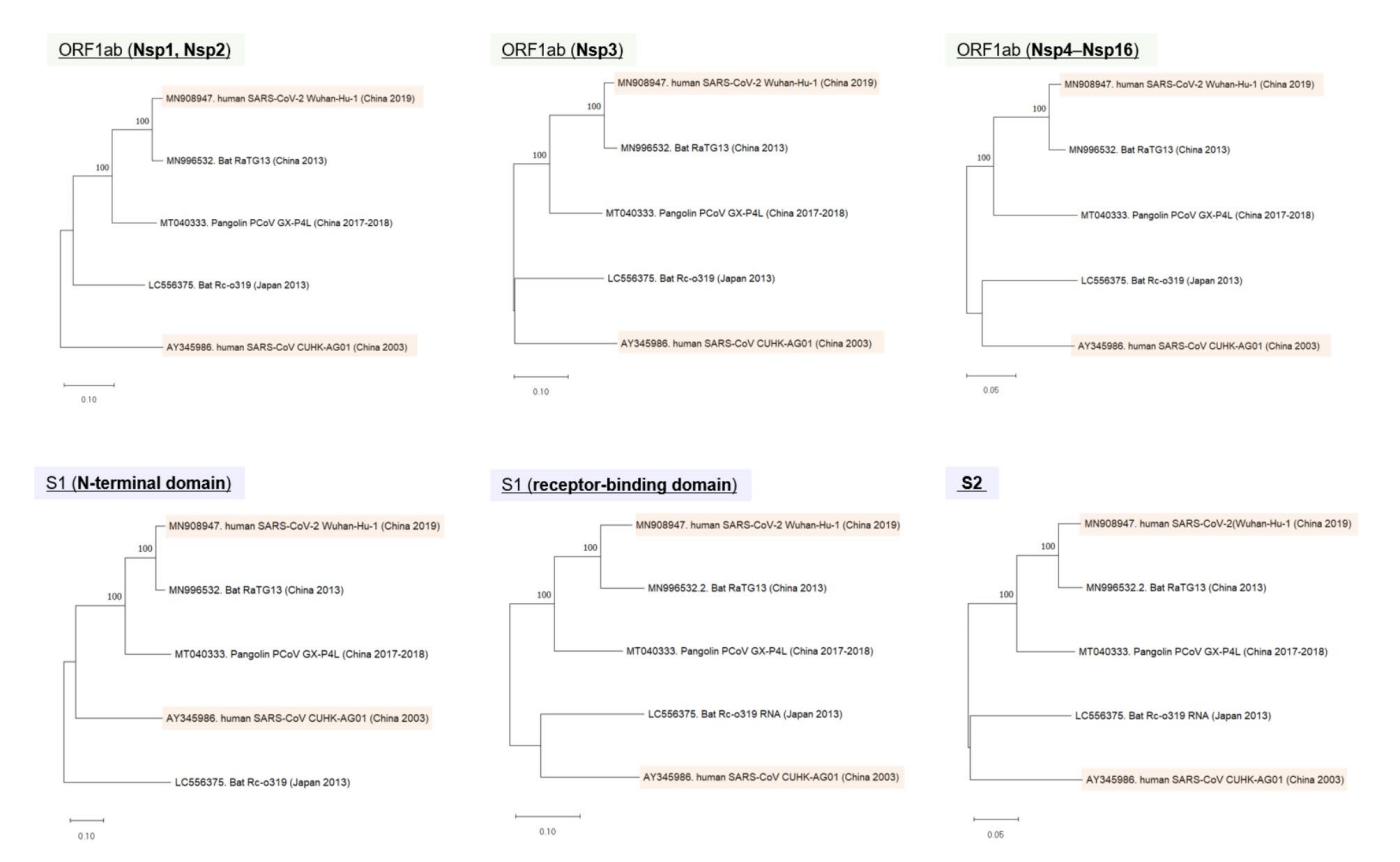
**Phylogenetic trees in specific genomic regions** To estimate ancestral states of the five evaluated SARS-related coronaviruses, gene region-specific phylogenetic trees with maximum likelihood (ML) method are inferred for the subdomains of ORF1ab gene (upper three) and spike gene (lower three). The numbers along the branches show the bootstrapping values indicating how many times out of 100 resampling is the same branch observed when repeating the phylogenetic reconstructions. The phylogeny study was performed using the MEGA 11 software Nsp, non-structural protein; ORF, open reading frame; S, spike; SARS-CoV, severe acute respiratory syndrome coronavirus

Finally, to estimate the ancestral state among the evaluated SARS-related coronaviruses, phylogeny study with SARS-CoV, SARS-CoV-2, RaTG13, Rc-o319, and GX-P4L genomes were performed by using maximum likelihood (ML) method. The inferred phylogenetic trees in different regions of ORF1ab and S genes are shown in Fig. 7. In all trees, RaTG13 (China, 2013) was the closest to SARS-CoV-2 (China, 2019), and GX-P4L (China, 2017–2018) aligned with the same clade with them. Meanwhile, SARS-CoV (China, 2003) and Rc-o319 (Japan, 2013) had divergent relationships with the other three viruses.

## Discussion

The present study demonstrated the presence of hotspots for indels in a broad spectrum of SARS-related coronaviruses from different animal hosts. Furthermore, both bat coronaviruses (RaTG13 from China and Rc-o319 from Japan) contain a large number of distinct indels based on a non-classical complex indels, which is called deletion-with-insertion mutation in the present study. This novel type of mutation replaces relatively long consecutive bases, often with changed base lengths. These non-classical complex indels apparently differ from each type of other conventional mutations, such as sole insertions, sole deletions, translocations, or variable numbers of tandem repeats [6, 7]. The abnormally high rates of point mutations and complex indels in the identified specific genomic regions suggest that the occurrence of mutations in these hotspots may be selectively neutral or even benefit the survival of the viruses through unknown mechanisms like antiviral immune evasion.

To date, the novel deletion-with-insertion mutation seems to be rare or even absent in human SARS-CoV-2 strains. Such a mutation or indel hotspot has not been reported in human SARS-CoV-2 strains in the last 2 years during the worldwide pandemic, although long insertions or deletions seldom occur in the human SARS-CoV-2 genome, such as the Δ382 variant (that is, 382 nucleotide deletion in ORF7b and ORF8 genes) from Southeast Asia in 2020 ^8^. This implies that the rate of indel occurrence in the human SARS-CoV-2 genome may be lower than that in bat or pangolin SARS-related coronaviruses, suggesting that the player of this novel type of mutation may originate from the host cells of non-human animal reservoirs, such as bats or pangolins, and may not be coded in the genomes of SARS-related coronaviruses. Further studies are needed to elucidate the potential roles of host animals in the occurrence of such high frequencies of indels in the genomes of SARS-related coronaviruses.

The exact mechanisms of such complex deletions-with-insertions in single-stranded RNA molecules are unknown. The clustered regularly interspaced short palindromic repeats-associated proteins (CRISPR-Cas) system enables the exchange of consecutive bases in double-stranded DNA molecules [9, 10], but an exchange of long consecutive bases in single-stranded RNA molecules is not feasible with the current CRISPR-Cas system alone [11]. Therefore, it is reasonable to assume that the observed long deletion-with-insertion mutations in RNA virus genomes were generated in natural environments through unknown molecular mechanisms. Another theory to explain the observed novel deletions-with-insertions may be the high frequency of single-nucleotide mutations close to the indel site, which has been previously reported in eukaryotes [12]. In this case, classical insertion or deletion, accompanied by frequent point mutations in the adjacent regions, may at least partially explain the observed novel type of mutations referred to as deletions-with-insertions in the present study. Future studies comparing genomes from larger numbers of SARS-related coronaviruses may clarify the developmental processes and molecular mechanisms of such unique mutations that create the indel hotspots in the Nsp3, S1, and ORF8 genes.

The present study has some limitations. First, whether the observed non-classical deletions-with-insertions was based on unknown novel developmental processes or can be explained by a combination of classical mutation types (that is, point mutations, insertions, or deletions) is uncertain. Alternatively, this novel type of indel may be explained only by the coincidental occurrence of classical insertions at the same base position in two different SARS-related coronaviruses. For example, if two viruses incorporate two different insertions from their common progenitor virus at the same base position, the mutation between these two viruses would appear to be based on a deletion-with-insertion. Although the probability of having many such coincidental phenomena across the genome is low, the establishment of a novel mutation type with deletions-with-insertions awaits validation through further research in the future. Second, although the Nsp2, Nsp3, S1, and ORF8 genes were identified as indel hotspots in the present study, the exact roles and functions of the encoded proteins of Nsp2, Nsp3 and ORF8 genes are currently unknown [13]. Consequently, the present study could not determine how frequent and complicated indels at the hotspots contributed to the survival and evolution of SARS-related coronaviruses in the past. Generally, most of the indels in the coding regions are deleterious for the survival of the viruses, and variants with such indels cannot compete with other variants and eventually become extinct. The abnormally high frequency of indels in these hotspot genes implies that mutations in these regions are typically neutral or even beneficial under positive selection. One possible explanation may be immune evasion based on the indels in these hotspots. Previous studies that evaluated SARS-CoV-2 epitope reactivity indicated that non-structural proteins such as Nsp3 and ORF8 may be highly antigenic T cell epitopes [14–16]. It is reasonable to consider that mutations in these genes may contribute to immune evasion by SARS-related coronaviruses. Further studies are required to elucidate the significance of indels in these hotspots for the survival and evolution of SARS-related coronaviruses. Finally, although this study estimated the ancestral state between the evaluated five viruses by generating phylogenetic ML trees, cautions are needed as the exact ancestral state among the five viruses is currently uncertain. Therefore, whether the observed non-classical complex indels have overlapped chronologically (as supposed in Fig. 4) or independently in the lineages of the bat and pangolin remains uncertain. Furthermore, the present study evaluated only five genomes. More number of virus species should be enrolled for generating phylogenetic trees to gain deeper and more reliable insights into the ancestral state.

In summary, the present study demonstrated the presence of indel hotspots in the Nsp2, Nsp3, S1, and ORF8 genes of SARS-related coronaviruses, as well as the presence of non-classical complex deletion-with-insertion mutations in these hotspots in a broad spectrum of SARS-related coronaviruses. Future research to elucidate the developmental mechanisms of such long indels, including complex deletions-with-insertions, would offer deeper insights into the process of viral evolution.

## Methods

### Sequences of the evaluated SARS-related coronaviruses

Genome sequences of SARS-CoV, SARS-CoV-2, and PCoV_GX-P4L were obtained from the NCBI GenBank database (https://www.ncbi.nlm.nih.gov/genbank/). The GenBank Accession ID for the human SARS-CoV CUHK-AG01 (China, 2003) was AY345986 [17], that for human SARS-CoV-2 Wuhan-Hu-1 (China, 2019) was MN908947 [18], that for bat SARS-related coronavirus RaTG13 (China, 2013) was MN996532.2 [19], that for bat SARS-related coronavirus Rc-o319 (Japan, 2013) was LC556375.1 [20], and that for pangolin SARS-related coronavirus PCoV_GX-P4L (China, 2017–2018) was MT040333 [21].

### Pairwise alignment and mutation classification

First, pairwise alignment of the complete genomes was manually performed between each pair of the evaluated SARS-related coronaviruses to obtain an overview of the distribution of indels across the genomes of SARS-related coronaviruses. The mutations were classified into the following two general types: point mutations and indels. Indels were further classified into classical simple insertions and deletions, and other complicated indels that cannot be grouped with classical insertions or deletions. The last indel type included indels with highly mutated sequences in bases around the gaps in aligned sequences, replacing the involved sequences to different ones with changed lengths. These non-classical complex indels were tentatively referred to as deletions-with-insertions in the present study. At each indel site, the possibility of other types of mutations, such as inversions or duplications, was examined. Based on the overall mutation rate of approximately 80%, gene sites with base substitutions of more than 10 bases in nearest ± 10 bases were considered potential indel sites, and the sequences were manually reviewed one at a time to avoid overlooking or overestimating the indels. The point mutation rates across the PCoV_GX-P4L genome are depicted as a line graph with a 95% confidence interval, and the rate at each base position is represented by the rolling average mutation rate in the nearest ± 50 bases.

### Multiple sequence alignment

The multiple sequence alignment process for the coding sequences of the five SARS-related coronaviruses was performed using Molecular Evolutionary Genetics Analysis Version 11 (MEGA11) software [22]. To align the sequences, the MUSCLE program was run with the following set of parameters: gap opening penalty score of -400 and gap extension penalty score of 0. The nearest sequences before and after each gap upon sequence alignment were reviewed to determine whether the observed gaps were derived from classical short in-frame indels or non-classical complex indels.

### Inference of phylogenetic ML trees in specific genomic regions

To estimate ancestral states among the evaluated five SARS-related coronaviruses, gene region-specific phylogenetic ML trees were inferred for different regions in ORF1ab (Nsp1–2, Nsp3, and Nsp4–16) and spike genes (N-terminal domain, receptor-binding domain, and S2), using Tamura-Nei model [23]. The phylogenetic trees were constructed by MEGA11 software with 100 bootstraps resampling. As the ML heuristic search process, nearest-neighbor interchange method was used. The branch lengths are proportional to the genetic distances measured by the number of substitutions per site.

## Data Availability

The genome sequences of SARS-CoV, SARS-CoV-2, and PCoV_GX-P4L were obtained from the NCBI GenBank database (https://www.ncbi.nlm.nih.gov/genbank/), with accession IDs AY345986, MN908947, and MT040333.
